# Effects of the age of vaccination on the humoral responses to a human papillomavirus vaccine

**DOI:** 10.1038/s41541-022-00458-0

**Published:** 2022-03-15

**Authors:** Francesco Nicoli, Barbara Mantelli, Eleonora Gallerani, Valentina Telatin, Laura Squarzon, Serena Masiero, Riccardo Gavioli, Giorgio Palù, Luisa Barzon, Antonella Caputo

**Affiliations:** 1 Department of Chemical, Pharmaceutical and Agricultural Sciences, University of Ferrara, 44121 Ferrara, Italy; 2 Department of Molecular Medicine, University of Padova, 35121 Padova, Italy

**Keywords:** Protein vaccines, Immunological memory

## Abstract

Adult vaccination programs are receiving increasing attention however, little is known regarding the impact of age on the maintenance of the immune response. We investigated this issue in the context of a human papillomavirus (HPV) vaccination program collecting real-world data on the durability of humoral immunity in 315 female subjects stratified according to vaccination age (adolescents and adults) and sampled at early or late time points after the last vaccine dose. HPV-specific IgGs, but not memory B cells, were induced and maintained at higher levels in subjects vaccinated during adolescence. Nonetheless, antibody functions waned over time to a similar degree in adolescents and adults. To shed light on this phenomena, we analyzed quantitative and qualitative properties of lymphocytes. Similar biochemical features were observed between B-cell subsets from individuals belonging to the two age groups. Long term humoral responses toward vaccines administered at an earlier age were comparably maintained between adolescents and adults. The percentages of naïve B and CD4^+^ T cells were significantly higher in adolescents, and the latter directly correlated with IgG titers against 3 out of 4 HPV types. Our results indicate that age-specific HPV vaccine responsiveness is mostly due to quantitative differences of immune cell precursors rather than qualitative defects in B cells. In addition, our results indicate that adults also have a good humoral immunogenic profile, suggesting that their inclusion in catch-up programmes is desirable.

## Introduction

Immunization programs for the pediatric population are usually well established, reaching coverage levels higher than those of adults^1^. However, the increase in life expectancy and the overall large number of older individuals in the Western population have highlighted the need of protecting the latter age group^2^. Adult immunization may nonetheless pose some challenges from the immunological point of view^3,4^.

Questions such as what is the best age to be immunized, the frequency of booster doses and whether age influences the declining rate of immune responses, are well addressed for pediatric vaccines, while for adults they are mostly based on empirical observations^4,5^. In this context, it is crucial to fully understand the kinetics of vaccine immunogenicity and the duration of immune responses as a function of age of immunization, especially for those vaccines intended to protect the adult population. HPV vaccination represents an ideal scenario, being routinely administered to adolescents and in catch-up programmes for adults.

The aluminum hydroxyphosphate sulfate salt-adjuvanted quadrivalent vaccine (4vHPV, Gardasil) against the human papillomavirus^6^ has been widely used for more than a decade, demonstrating very high levels of efficacy^7,8^ and relying mainly on neutralizing antibodies^9–11^. Infection with high-risk HPV types, mainly HPV-16 and HPV-18, is associated with the occurrence of ano-genital and oropharyngeal precancerous lesions and cancers, and infection with low-risk HPV-6 and HPV-11 is a common cause of genital warts. These conditions, which represent a relevant disease burden worldwide, can be prevented by vaccination. After an initial focus on adolescents, current HPV vaccination campaigns also include adults. However, in general immune system development occurs in the first years of life until adolescence and the changes in the immune functions observed between teenagers and adults, suggest that similar interventions (e.g. immunization programs) may require adjustments depending on the target age group. Indeed, the same vaccine may have different outcomes depending on the age of the recipient.

In this study we aimed to understand the impact of age on the maintenance of the immune response, by investigating different aspects of humoral immune responses at short and long time intervals subsequent to vaccination with 4vHPV in women of different age groups. Moreover, we also investigated the persistence of immunity in the adult population following childhood vaccination in the first year of life, by assessing antibody levels.

## Results

### Vaccination during adolescence induces high and long-lasting 4vHPV-specific IgG responses

**Fig. 1:**
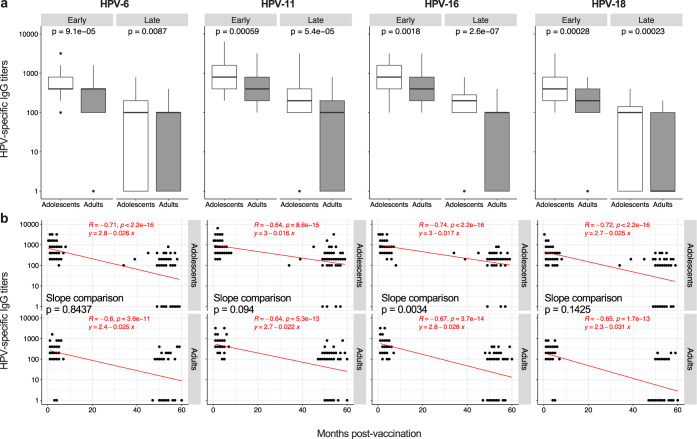
Induction and maintenance of HPV-specific IgG. **a**, **b** HPV-6, HPV-11, HPV-16, and HPV-18-specific IgG titers measured by ELISA are shown as box and whisker plots (**a**) and correlated with time (months) post-vaccination (**b**); *n* = 60 and *n* = 59 for adolescents of the “Early cohort” and the “Late cohort”, respectively; *n* = 45 and *n* = 56 for adults of the “Early cohort” and the “Late cohort”, respectively. Statistical significance was determined by the Mann–Whitney (**a**) and Spearman’s rank correlation (**b**) and all *p*-values are shown. The box and whisker plots display the median (center line), the first and third quartiles (the two hinges) and two whiskers: the upper whisker extends to the largest value no further than 1.5 × inter-quartile range (IQR) from the hinge while the lower whisker extends from the hinge to the smallest value at most 1.5 × IQR of the hinge. Data beyond the end of the whiskers are plotted individually. The equation of the regression lines has to be intended as: Log(y) = q + mx, and the comparison between slopes (adolescents vs adults) was calculated by ANCOVA (**b**). “Early cohort”, Early; “Late cohort”, Late.

To determine whether the age of vaccination influences the induction and the duration of the immune response to the 4vHPV vaccine, HPV-specific antibodies were measured 1–8 months (“Early cohort”) and 1–5 years (“Late cohort”) after immunization in female subjects stratified in two groups based on the age at the time of vaccination (Table 1): adolescents (10–14 y) and adults (18–53 y). As shown in Fig. 1a, HPV-specific IgG titers were significantly lower in adults than in adolescents for both the “Early cohort” and “Late cohort”. This was true for each HPV vaccine type, and indicates that the age of vaccination affects both primary and long-term responses.

**Table 1:** Cohort description.

Cohort	Age group	*N* =	Age (median; range)	% of females	% of participants with completed vaccination course (3 doses^a^)	Month of blood drawing after the third vaccine doses (median; range)
Early cohort	Adolescents	117	11 y; 10–14 y	100	100	2; 0.3–8
	Adults	57	26 y; 18–53 y	100	100	3; 1–7
Late cohort	Adolescents	70	12 y; 11–14 y	100	100	53; 17–59
	Adults	71	21 y; 18–26 y	100	100	52; 12–60

^a^The vaccine has been administered by intramuscular route. Boosting doses were given at 2 and 6 months after the priming dose.

The magnitude of memory immune responses is usually correlated with that of primary responses^12^. However, the survival of long-lived lymphocytes may also be influenced by several intrinsic and extrinsic factors, thus affecting memory maintenance irrespectively of primary responses^13,14^. We thus sought to investigate whether the lower long-term humoral responses observed in the “Late cohort” in adults compared to adolescents was related to the influence of age on primary responses, on the persistence of memory responses or on both. To this aim, we evaluated the time-dependent decrease of 4vHPV-specific IgG in the two age groups measuring the correlation between antibody titers and time (in months) after vaccination. The slope of the regression lines, which indicate how the variables (“months post-vaccination” and “IgG titers”) are related, was compared between adolescents and adults. As expected, in both age groups we observed a loss over time of vaccine-specific IgG (Fig. 1b). The slope of the regression lines was significantly steeper in adults than in adolescents for HPV-16. A similar trend (steeper regression line in adults), although not significant, was observed also for HPV-11 and HPV-18. On the other hand, the decay of HPV-6-specific IgG was comparable between the two age groups. This indicates that the increase in the age of vaccination is associated with a faster loss of circulating antibodies. However, this occurs only against specific HPV subtypes, suggesting that other confounding factors (e.g. natural exposure) could contribute to this phenomenon.

Overall, these data suggest that both lower vaccine-induced primary responses and a more rapid IgG decline contribute to the inferior long-term antibody titers in adults compared to adolescents.

### Vaccination during adolescence induces highly functional 4vHPV-specific IgG responses

**Fig. 2:**
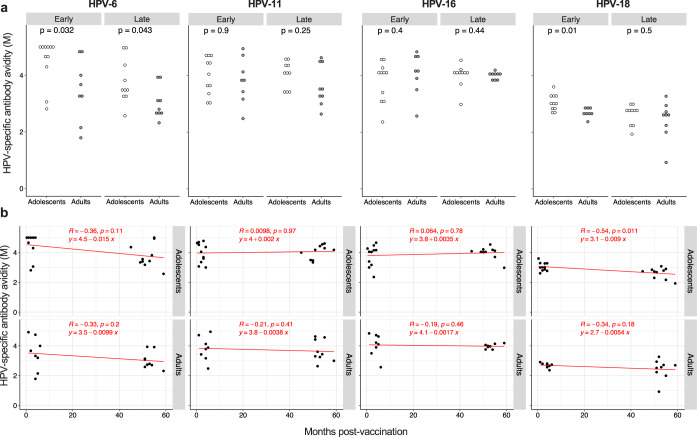
Effect of age on the avidity of HPV-specific antibodies. **a** Avidity of HPV-6, HPV-11, HPV-16, and HPV-18-specific specific IgG, measured in a subgroup of ELISA responders, reported as molarity (M). Each dot represents a single donor; *n* = 11 and *n* = 10 for adolescents of the “Early cohort” and the “Late cohort”, respectively; *n* = 8 and *n* = 9 for adults of the “Early cohort” and the “Late cohort”, respectively. **b** Molarity values (M) were correlated with with the months post-vaccination. Statistical significance was determined by the Mann–Whitney (**a**) and Spearman’s rank correlation (**b**) and all *p*-values are shown. “Early cohort”, Early; “Late cohort”, Late.

The IgG avidity index for each HPV vaccine type was determined in a subset of vaccinees. As shown in Fig. 2a, the avidity of antibodies specific for HPV-6 was lower in adults compared to adolescents in both the “Early cohort” and “Late cohort”. The same was observed for HPV-18-specific IgG, although only in the “Early cohort”. The HPV-11 and HPV-16 avidity indexes were comparable in the two age groups of both the “Early cohort” and the “Late cohort”. A decay over time after vaccination of antibody avidity was generally not observed in either age-group (Fig. 2b), with the exception of HPV-18-specific IgG in adolescents. Thus, the age of vaccination affects the induction of high avidity antibodies only in respect to two HPV subtypes and does not influence their time-dependent fluctuations.

**Fig. 3:**
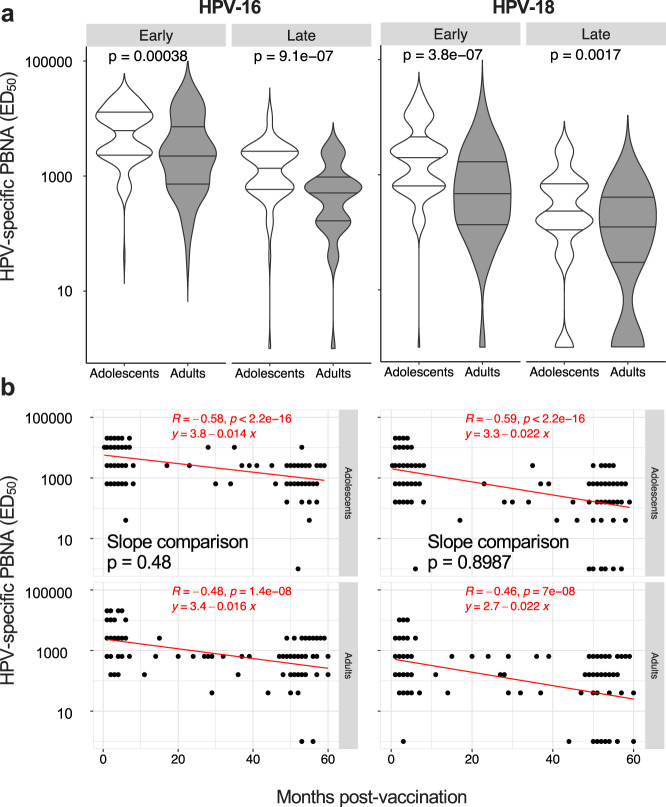
Induction and maintenance of HPV-specific neutralizing antibodies. **a**, **b** HPV-16 and HPV-18-specific specific nAbs were measured by the PBNA, reported as ED_50_, shown as violin plots (**a**) and correlated with the months post-vaccination (**b**); *n* = 116 and *n* = 70 for adolescents of the “Early cohort” and the “Late cohort”, respectively; *n* = 57 and *n* = 71 for adults of the “Early cohort” and the “Late cohort”, respectively. Statistical significance was determined by the Mann–Whitney (**a**) and Spearman’s rank correlation (**b**) and all *p*-values are shown. The lines in the violin plots represent the median and the IQR, while the width of violins represents the density (frequency of values). The equation of the regression lines has to be intended as: Log(y)=q+mx, and the comparison between slopes (adolescents vs adults) was calculated by ANCOVA (**b**). “Early cohort”, Early; “Late cohort”, Late.

We next assessed the neutralization capacity of HPV-specific antibodies. In line with the age drop of vaccine-induced IgG (Fig. 1), neutralizing antibodies against HPV-16 and HPV-18 (PBNA titers) were significantly lower in adults compared to adolescents in both the “Early cohort” and “Late cohort” (Fig. 3a). However, the reduction of PBNA titers over time after vaccination occurred to similar degrees in the two age groups, as suggested by the non-significantly different slopes of the regression lines calculated between months post-vaccination and neutralizing antibodies in adolescents and adults (Fig. 3b).

These results suggest that adults may develop less functional antibodies compared to adolescents upon 4vHPV vaccination. However, while IgG neutralization capacity drops with time independently of the age of vaccination, antibody avidity remains stable up to 5 years after vaccination.

### The age of vaccination influences the maintenance but not the induction of memory B cells

**Fig. 4:**
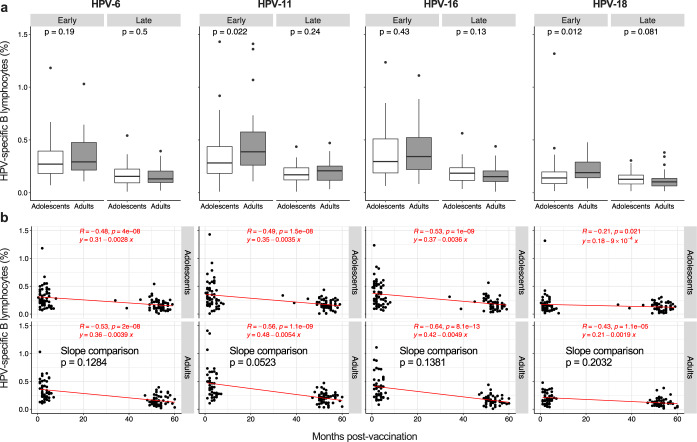
Induction and maintenance of HPV-specific memory B lymphocytes. **a**, **b** HPV-6, HPV-11, HPV-16, and HPV-18-specific memory B lymphocytes measured by B cell Elispot assay are shown as box and whisker plots (**a**) and correlated with the months post-vaccination (**b**); *n* = 58 and *n* = 59 for adolescents of the “Early cohort” and the “Late cohort”, respectively; *n* = 44 and *n* = 56 for adults of the “Early cohort” and the “Late cohort”, respectively. Box and whisker plots are as in Fig. 1. Statistical significance was determined by the Mann-Whitney (**a**) and Spearman’s rank correlation (**b**) and all *p*-values are shown. The comparison between slopes (adolescents vs adults) was calculated by ANCOVA (**b**). “Early cohort”, Early; “Late cohort”, Late.

We next measured the amount of vaccine-specific memory B cells in our cohorts. Unexpectedly, their frequency was not affected by the age of vaccination (Fig. 4a), with the exception of HPV-11 and -18 specific B cells. For these HPV types, we observed higher responses in adults of the “Early cohort”. However, these differences were lost in the “Late cohort”, suggesting that adults experience a greater loss of memory B cells over time, as observed for antibodies. Indeed, the slopes of the correlation lines between months post-vaccination and percentages of HPV-specific memory B cells were generally steeper in adults, although these differences never reached statistical significance (Fig. 4b).

Taken together, these results indicate that the 4vHPV vaccine-induced similar frequencies of memory B cells in adolescents and adults. This behavior was different from that of circulating IgG antibodies, which were induced to lower levels with increasing vaccination age, even in primary immune responses.

### Vaccine-specific humoral responses are comparably induced between young and middle-aged adults

Our data suggest that the age of vaccination primarily impacts the induction of 4vHPV-specific IgG (binding and neutralizing), whose titers are higher in adolescents compared to adults. To assess whether this paradigm is confirmed also along adulthood, we compared humoral responses to the 4vHPV between young adults (18–26 y) and middle-aged adults (27–53 y) in the “Early cohort” (see Supplementary Fig. 1 for age distribution).

**Fig. 5:**
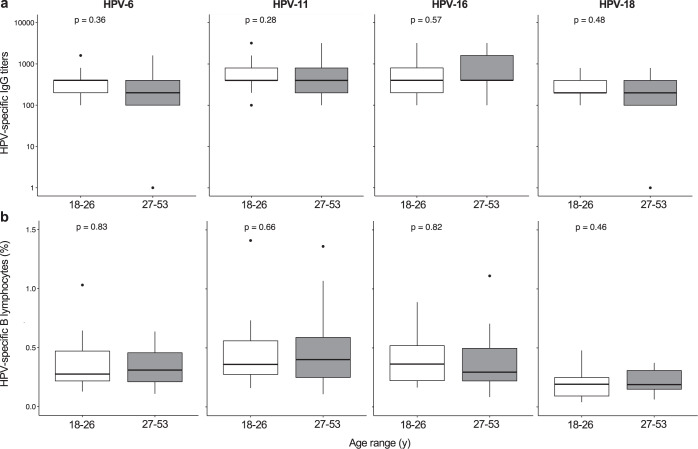
Induction and maintenance of HPV-specific IgG and memory B lymphocytes in adults. **a**, **b** HPV-6, HPV-11, HPV-16, and HPV-18-specific IgG titers measured by ELISA (**a**) and memory B lymphocytes measured by B cell Elispot assay (**b**) are shown as box and whisker plots after stratification of adults for age range: 18–26 y and 27–53 y; *n* = 21 for younger adults and *n* = 23 for middle-aged adults. Box and whisker plots are as in Fig. 1. Statistical comparisons were made the Mann–Whitney test and all *p*-values are shown.

For each HPV subtype, both IgG (Fig. 5a) and neutralizing antibody titers (Supplementary Table 1) were comparable between 18–26 y and 27–53 y subjects. In addition, the frequency of HPV-specific memory B cells was similar between young and middle-aged adults (Fig. 5b).

Thus, our data suggest that the passage from adolescence to adulthood represents a turning point for the subsequent decline of primary immune responses.

### Humoral responses to childhood vaccines are maintained to similar levels in young and middle-aged adults

**Fig. 6:**
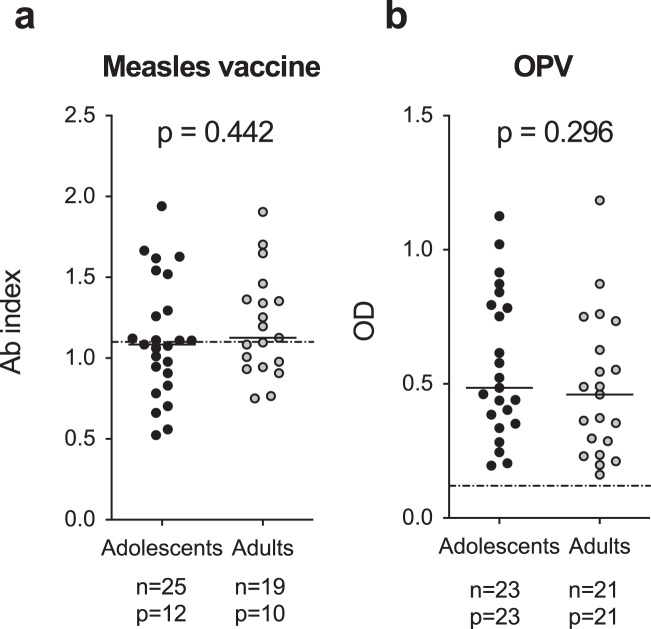
Humoral immune responses toward childhood vaccines. **a**, **b** The presence of antibodies toward the measles (**a**) and oral polio (OPV, **b** vaccines were measured in subjects and presented as antibody index or optical density (OD), according to the assay used. Dotted lines represent cut-off values for positivity. The number of subjects analyzed (n) and of positive individuals (p) for each age-group are reported in the figure. Statistical significance was determined by the Mann-Whitney test and all *p*-values are shown.

Our data show that long-term HPV-specific IgG are maintained less in adults as a result of a poorer induction of primary responses and also because of a faster decay. This latter aspect suggests problems at the level of long-lived antibody-secreting cells. We wondered if these age-related defects could embrace the whole long-lived antibody-secreting cell compartment or only those specific for HPV. In the first case, the ageing process should affect all previously established antibody-secreting cells, with lower levels of long-term humoral immunity against previously encountered antigens. In the second case, only antibody-secreting cells generated later in life (adulthood vs adolescence) should be affected, and thus only HPV-specific- IgG responses should be reduced. To investigate this issue, we measured humoral responses toward the measles and oral polio (OPV) vaccines, which were given to both adolescents and adults in their first year of life. As shown in Fig. 6, responses were comparable among the two age groups. In particular, about half of adolescents and adults have detectable anti-measles IgG (Fig. 6a) and all individuals possess anti-OPV IgGs (Fig. 6b). No correlations were observed between the responses toward these 2 vaccines and the 4vHPV-specific IgG titers (Supplementary Table 2). Collectively, these results exclude an age-dependent deterioration of the whole compartment of long-lived antibody-secreting cells in adulthoods.

### Quantitative but not qualitative alterations of naïve B and CD4^+^ T cells are detected with increasing age

To investigate whether the age of vaccination affects the quantity and quality of plasma cells (PC) responsible for the secretion of circulating antibodies, we measured their frequency and metabolic properties, in the “Early cohort”. However, no significant differences were observed between adolescents and adults (Supplementary Fig. 2), nor did we observed a correlation between the percentages of PC and HPV-specific IgG titers (Supplementary Table 3).

Consistent with the similar establishment and maintenance of HPV-specific memory B cells in the two age groups, total memory B cells did not show any age-related biochemical differences (Supplementary Fig. 3a), nor did we observed a correlation between the percentage of memory B cells and HPV-specific IgG titers (Supplementary Table 3).

**Fig. 7:**
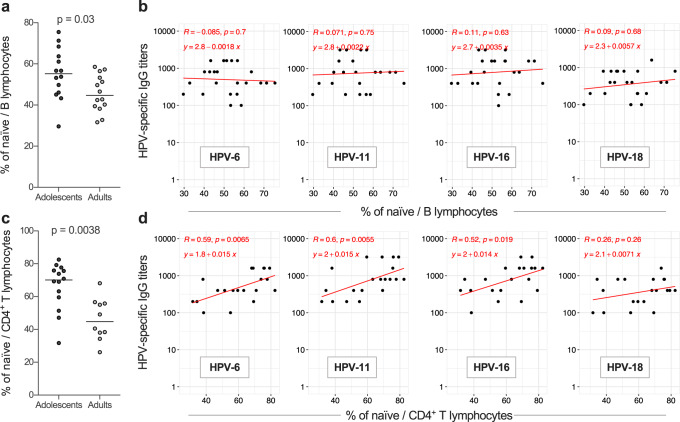
Effect of age on the quantity of naïve lymphocyte subsets. **a**, **b** Percent of naïve B cells on total B lymphocytes (**a**, each dot represents a single donor and lines the median) and their correlation (**b**) with HPV-6, HPV-11, HPV-16 and HPV-18-specific IgG titers (regression lines and their equations are shown); *n* = 28 (14 adolescents and 14 adults). **c**, **d** Percent of naïve CD4^+^ T cells on total CD4^+^ T lymphocytes (**c**, each dot represents a single donor and lines the median) and their correlation (**d**) with HPV-6, HPV-11, HPV-16, and HPV-18-specific IgG titers (regression lines and their equations are shown); *n* = 24 (14 adolescents and 10 adults). Samples are from the “Early cohort”. Statistical significance was determined by the Mann–Whitney test and Spearman’s rank correlation and all *p*-values are shown.

Stronger primary humoral responses and slower loss of immune memory may also depend on the priming of naïve B lymphocytes which, in turn, is affected by their basal properties. However, no specific age-related biochemical patterns in naïve B cells were identified (Supplementary Fig. 3b). We next evaluated if the amount of naïve B cells could impact the vaccine response. Indeed, as already observed for CD8^+^ T cells, a low number of precursors could negatively impact primary responses^15^. Our results show that the percentage of naïve B cells was significantly higher in adolescents than in adults (Fig. 7a), but did not correlate with IgG titers in the “Early cohort” (Fig. 7b). Of interest, in adults a significant decline in naïve CD4^+^ T cells which are key players in the induction of humoral responses, was observed (Fig. 7c). Furthermore, the percentage of naïve CD4^+^ T cells directly correlated with IgG titers against 3 out of 4 HPV vaccine types (Fig. 7d). Collectively, these data suggest that adolescents benefit of a stronger CD4^+^ T-cell mediated immune response which boosts their short- and long-term humoral response upon vaccination. Therefore, age-specific patterns of vaccine responses are mostly due to quantitative differences of immune cell precursors rather than defects in plasma cells or memory B cells.

## Discussion

Our data show that adolescents respond better than adults to the 4vHPV vaccine, as they present higher levels of both IgG titers and neutralizing antibodies. These findings confirm previous reports showing higher vaccine-induced HPV-specific humoral responses (both binding and neutralizing) in adolescents compared to young adults at short time-points after vaccination^16–21^. Such an effect has been previously demonstrated with other vaccines, including those to Ebola virus^22^ and hepatitis B virus^23^. Of note, in the latter case, an age cut-off of 30 years was suggested, as older individuals showed a greater proportion of nonresponders^23^. Interestingly, the 4vHPV vaccine was shown to have comparable immunogenicity in 9–10- and 11–13-year-old girls^24^, thus indicating that the age-dependent decrease of vaccine-induced humoral response starts after adolescence^20^. It should be noted however that despite the observed lower antibody titers in adults than adolescents, seroconversion was fully achieved in all age groups shortly after vaccination^16–19,21^.

Interestingly, the persistence of binding antibodies was superior in younger vaccine recipients. Although we cannot exclude that sexual debut occurring in adolescents may have boosted vaccine responses in this age-group, the increased humoral responses in younger vaccine recipients has been observed for other vaccines as well including the bivalent HPV-16/HPV-18 vaccine Cervarix^25^ and the smallpox-vaccine^26^. In the latter context, children-recipients maintained humoral immunity for decades while the majority of adults-vaccinees have undetectable antibody levels within 10 years^26^. Nonetheless, it should be noted that in the case of 4vHPV vaccination, protective levels of binding and neutralizing IgG are maintained in adults of different age-groups (ranging from 18- to 45-year-old) for several years after vaccination^27^. This is particularly true for nAb, whose physiological drop over time has been shown to be age-independent^18,19^. Consistently with the concept that age of vaccination has mild effects on antibody quality, we observed subtle and scarce differences in antibody avidity between adolescents and adults. Similar findings have been observed in other studies comparing the avidity and affinity of IgG induced by inactivated influenza virus vaccines in subjects ranging from 18- to 100-year-old^28^ or by tick-borne encephalitis virus vaccines in subjects <30 y and >50 y^29^.

As we could not measure pre-vaccination HPV seroprevalence, we are unable to ascertain at which level pre-existing immunity may have influenced our results. However, a recent study has shown that vaccine-induced IgG levels are comparable between subjects that were HPV seropositive or seronegative at the time of vaccination^30^. This seems to be caused by low titers, avidity and neutralizing capacity of antibodies induced by natural infections^31,32^. It is thus very unlikely that the lower responses we observed in adults could be due to pre-existing IgGs that neutralize the VLPs and hamper vaccine immunogenicity. Rather, we would expect the inverse to occur, such as a higher immunogenicity in HPV seropositive women due to the boosting of pre-existing immunity. Nevertheless, this phenomenon does not seem to occur, or rather contributes marginally to humoral responses in adults (which should have more chances, compared to adolescents, of a previous exposure to HPV).

Surprisingly, we observed that vaccine-specific memory B cells were induced at similar, or even slightly higher levels in adults compared to adolescents. In agreement with our results, Smolen and colleagues^33^ showed that HPV-6-, HPV-11- and HPV-16-specific memory B cells measured 1 month after the last dose, were similar in 9–13-year-old and 16–26-year-old subjects vaccinated with the 4vHPV vaccine; in contrast to our observations however, the younger age group developed higher numbers of HPV-18-specific memory B cells. The frequencies of memory B cells observed in our study were in general higher than those reported by Smolen et al.^33^. Such an effect may be caused by methodological differences (i.e. at the level of Elispot procedures) between the two studies. Further differences may be accounted for by the fact that we collected samples at time points beyond 1 month after the last dose, and the fact that we also included subjects that were older than those enrolled by Smolen et al. Our study was performed in a real-world context, and thus we might have included also HPV-seropositive women. As their proportion is known to increase with age^17,34^, we cannot exclude that the higher frequency of HPV-specific memory B cells observed for some HPV vaccine types is due to a secondary response and thus proliferation of this cell subset. However, the HPV seroprevalence in the Italian unvaccinated women population is around 8%^35^, and this percentage is usually higher in younger compared to middle-aged adults^36,37^. Therefore, the relative low proportion of seropositive women in the unvaccinated Italian population and the similar percentages of HPV-specific memory B cells that we measured between 18–26 y and 27–53 y adults would argue against a major role of pre-existing memory B-cells in the peculiar age-specific pattern we observed, though further studies are needed to confirm this aspect. Consistently, in our cohort, adults displayed a higher percentages of memory B cells specific for HPV-11 and HPV-18 and not for HPV-6 and HPV-16 compared to adolescents. If this was due to a previous exposure, we would expect the inverse to occur, with adults presenting higher responses toward HPV-6 and HPV-16 since the latter are more than two times more prevalent than HPV-11 and HPV-18^36,37^.

Of note, the phenomenon of similar frequencies of memory B cells despite different antibody levels between two distinct age groups has been observed also for other vaccines^38^. Frasca and colleagues^38^ observed that age does not affect the percentages of vaccine-induced circulating PC. We also observed comparable PC frequencies between adolescents and adults in the “Early cohort”, although our analysis focused on bulk, and not antigen-specific, PC. In addition, we failed to find correlations between circulating PC and HPV-specific IgG. Thus, although further analyses are required, we could speculate that the lower long-term vaccine-specific IgG we observed in adults is not due to alterations of the circulating PC compartment. We would also tend to exclude age-related issues of PC replenishment by memory B cells, as vaccine-specific memory B lymphocytes were comparable among age groups and, more in general, circulating memory B cells and antibodies are thought to be independently regulated^39,40^. More in-depth investigations are needed to assess any potential age-related defect during the generation of long-lived non-circulating plasma cells (LLPC). The comparable responses toward childhood vaccines that we observed in the two age-groups would exclude an age-dependent decline of this compartment (i.e. previously established LLPC), while it is more plausible that their induction is altered with increasing age.

The numbers of naïve B cells decreases with age^41^. It has been proposed that a reduction in the number of vaccine-specific PC (and thus of naïve B cells primed by the vaccine antigen), and not a reduction of secreted IgG by PC, explains the lower humoral responses with advancing age^28,42^. Despite the observation that the B-cell compartment was shifted toward memory phenotypes in our cohorts of adult individuals, the frequency of naïve B cells did not correlate with HPV-specific IgG. This might also be due to the fact that we did not measure absolute numbers of naïve B cells, but their percentage. In addition, it has been proposed that increasing age is associated with a lower proliferative capacity of B cells^41^, thus suggesting that the higher numbers of vaccine-specific PC observed by others in young recipients^28,42^ may be due to both higher precursor numbers and their increased ability to generate antibody-secreting cells. So far, few promising studies have characterized age-associated metabolic changes at the B-cell level^43^. However, in this respect, we did not observe any difference between adolescents and young adults. Nonetheless, biochemical perturbations could occur later in life and thus be undetectable in our cohorts which did not include elderly individuals.

Finally, we observed a correlation between naïve CD4^+^ T cells and HPV-specific IgG. Naïve CD4^+^ T cells will differentiate into different types of effectors, including T follicular helper (TFH) cells, upon interaction with their cognate antigen. Such CD4^+^ T cells are important for the generation and maturation of antibody-secreting cells^44,45^. The interaction between naïve B cells and TFH cells occurs within the germinal centers^43^ and is defective in aging, leading to a reduced humoral responses^46,47^. Therefore, lower numbers of naïve CD4^+^ T cells could result in lower humoral responses.

This study has some limitations. In particular, the lack of data about HPV natural exposure prior to vaccination and/or sampling and about behavioral attitudes (i.e. sexual activity) may hamper result interpretation. Nonetheless, the study was intended to collect real-world data, and all the biases that could have affected our results will be, most likely, also present at a population level. This possibly makes our findings generalizable within the HPV-vaccine context.

In conclusion, modern vaccinology recognizes the importance of immunization programs for young and middle-aged adults, as the major burden of vaccine-preventable infections (VPIs) is in adults^2,48,49^ who, together with adolescents, constitute a population often affected by VPI outbreaks^50,51^. Furthermore, adults can be a source of contagion for more fragile individuals (e.g. newborns or elderly)^52^. As a consequence, a growing number of countries is implementing vaccine campaigns for adults. In this study, we observed that adults, although displaying lower responses than adolescents, present with detectable and well maintained humoral responses in particular with respect to memory B cells after HPV vaccination. Our study provides encouraging results of vaccination programs aimed at embracing all age groups. Nonetheless, when applicable, vaccinations at younger age (e.g. adolescence) are still the gold standard to achieve high personal protection levels. In particular, our data strongly support the large-scale implementation of HPV immunization programs for teenagers.

## Methods

### Study subjects and sample purification

This observational, cross-sectional study was conducted in the framework of an organized vaccination program in Italy. We evaluated and compared the immune responses to the 4vHPV vaccine (Gardasil, Merck) in 315 female subjects. One subgroup (*n* = 174) was tested at 1–8 months (median of 2 months) after the third vaccine dose and is defined as “Early cohort”, while a second subgroup (*n* = 141) was tested at 1–5 years (median of 4 years) after the third vaccine dose and is defined as “Late cohort”. In the “Early cohort”, 117 were adolescents at the time of vaccination (age range: 10–14-year-old, median age: 11 y) and 57 were adults (age range: 18–53-year-old, median age: 26 y). In the “Late cohort”, 70 were adolescents at the time of vaccination (age range: 11–14-year-old, median age: 12 y) and 71 were adults (age range: 18–26-year-old, median age: 21 y).

Enrollment was carried out at the public health district of Padova (Veneto region, Italy) where HPV vaccine was offered by organized vaccination (10–14 years old) and implementation (older ages) programs. Girls and women who had previously completed the vaccination schedule were invited by letter to participate in the study, which consisted in blood sampling, to measure the persistence of the immune response elicited by the vaccination. Exclusion criteria were fever and other symptoms of active infection in the three weeks prior to blood collection, presence of chronic inflammatory diseases, immunosuppressive therapy, and pregnancy. The study was approved by the Ethics Committee of Padova University-Hospital (Protocol N° 2413P) and conducted according to the principles expressed in the Declaration of Helsinki. All participants, or their parents for minors, signed a written informed consent form.

A control cohort of 24 unvaccinated women with unknown previous history of HPV infection (age range: 22–40-year-old) was recruited and samples analyzed to determine the cut-off values of immunogenicity assays.

Peripheral blood mononuclear cells (PBMCs), plasma and sera were purified from whole blood for subsequent analyses by Ficoll-Paque (GE-Healthcare, Milan, Italy) density gradient centrifugation (for PBMCs and plasma specimens)^53^ and serum separator tubes (for serum specimens). Not all the assays were performed on all subjects. The number of individuals tested for each analysis is specified in the Figure legends.

### VLPs preparation

Plasmids p6sheLLr+, p16sheLL, and p18sheLL, containing L1/L2 genes of HPV6, 16 and 18 were kindly provided by dr. John T. Schiller (NIH, Bethesda, USA), whereas plasmid p11L1h containing the L1 gene of HPV11 was kindly provided by dr. Martin Müller (German Cancer Research Center, Heidelberg, Germany). The preparation and purification of HPV type-specific virus like particles (VLPs) was done according to the protocol of Buck et al.^54^ in 293TT cells^53^. The 293TT cell line was kindly provided by John T. Schiller (NIH, Bethesda) and then grown in Dulbecco’s modified Eagle’s Medium (DMEM) (Gibco Life Technologies) containing 10% FBS, 1% antibiotic-antimicotic solution, 1% glutamax, 1% non-essential aminoacids (all from Gibco Life Technologies), and 250 μg/mL hygromycin B (Sigma-Aldrich). For each HPV type-specific VLP, 293TT cells (7 × 10^6^) were seeded in 75 cm^2^ flasks and incubated 16–18 h at 37 °C up to 50% confluence. Cells were lipofected (Lipofectamin 2000, Invitrogen) with 20 μg of plasmid DNA, according to manufacturer’s instructions, which were incubated at 37 °C and then collected 48 h later. The cells were washed in Dulbecco’s phosphate buffered saline (D-PBS) containing 9.5 mM MgCl_2_, transferred to new eppendorf tubes and centrifuged at 2000 rpm (Centrifuge 5418 R, Eppendorf) for 7 min. The cellular pellet was resuspended in D-PBS (1.5× the cellular volume) and then lysed with Triton X-100 (1/20 of the volume), 0.1% Benzonase (Sigma–Aldrich, Milan, Italy) 0.1% Plasmid Safe (Epicentre, Madison, WI, USA), and 1 M ammonium sulfate (pH 9) (1/40 of the volume) at 37 °C for 24 h. The cell lysate was added with 0.17 M NaCl, incubated on ice for 20 min. and centrifuged at 10,000 rpm at 4 °C. The supernatant was left on ice for two hours and VLPs were purified by means of an iodixanol gradient (Sigma–Aldrich) at 50,000 rpm for 3.5 h (rotor SW55ti, Beckman Coulter, Milan, Italy). Fractions were collected from the bottom of the tube, read for protein concentration (BCA protein assay kit, Pierce, Milan, Italy), and stored at −80 °C until use. The fractions were analyzed by SDS-PAGE, silver staining (Silver stain kit, Pierce) and western blot using mouse anti-L1 primary IgG antibodies (HPV6/11: Antibodies-online; HPV16: Santa Cruz Biotechnology, Dallas, Texas; HPV18: Abcam, Cambridge, UK), and an anti-mouse HRP-secondary antibody (Abcam). For each type-specific VLP, all of the fractions positive for L1 derived from several transfections and purifications procedures were pooled together to obtain a single type-specific VLP stock to be used for all immunological assays (IgG Elisa, avidity index, B-cell Elispot). The protein concentration of each VLP stock was measured by BCA and disposable aliquots (25 μg) were stored at −80 °C until use. All of the plasticware used in these procedures was siliconized (StarLab, Milan, Italy).

### Neutralization assay

The preparation and purification of HPV type-specific pseudovirions (PsVs) encapsidating a SEAP reporter plasmid and the neutralization assays on serum samples were done in 293TT cells^55^. PsVs were obtained after transfection of cells with equal amounts of p16shell, p18shell, p31shell, or p45shell plasmids and the reporter pYSEAP plasmid. The neutralization assays were done on cells seeded in 96-well plates at a concentration of 30,000/well in assay buffer [DMEM without phenol red, 10% of FBS (Lonza, Basel, Switzerland), 1% of Glutamax (Gibco Life Technologies, Thermo Fisher Scientific, Monza, Italy), 1% of non-essential amino-acids], grown for 2–5 h at 37 °C and incubated with HPV PsVs alone or previously mixed with sera (two-fold dilution from 1:40 to 1:163,840) in duplicate wells. After 72 h, SEAP expression was measured in cell supernatants using the SEAP Reporter Gene Assay. The neutralizing titer was determined as the reciprocal of the final dilution of serum that yielded <50% of mean RLU measured with PsVs alone and reported as ED50 (effective dose producing 50% response). The limit of quantification of the assay was set at 40 ED50. Samples with neutralizing titers equal to or higher than 40 ED50 were considered positive, whereas samples with neutralizing titers lower than 40 ED50 were assigned a value of 1 for the purpose of geometric mean titer (GMT) calculation.

### HPV IgG titers and avidity and measles and OPV antibodies

HPV type-specific IgG titers were measured by ELISA^53^. Plates were coated with 100 μl/well of each HPV type-specific VLP at 4 °C for 18–20 h, washed and incubated with 1% bovine serum albumin (BSA, Sigma-Aldrich, Milan, Italy) in PBS for 1 h and then with serially diluted (1:2 dilution) plasma samples (50 μl) (duplicate wells) for 2 h at 37 °C. After extensive washing, immunecomplexes were detected by incubation with a goat anti-human IgG-HRP (Abcam, Cambridge, UK) for 1 h and addition of ABTS substrate (Thermo Fisher Scientific) for 30 min at 37 °C. In each plate, blank (wells with PBS-1% BSA alone), negative controls (wells with a pool of plasma of unvaccinated subjects), and positive controls (wells with a pool of plasma of vaccinated subjects) were included. Optical density (OD) at 620 nm was determined with an automatic plate reader and each OD value was subtracted from the blank^12,56^. Titers were determined based on the reciprocal of the dilutions interpolating the cut-off value. For each type-specific VLP, the cut-off values were 0.374 for HPV-16; 0.346 for HPV-18; 0.388 for HPV-6; 0.346 for HPV-11^53^, and were calculated as the mean +3 standard deviations of the OD values of the control cohort.

Measles- and OPV- specific antibody presence was determined on plasma samples using commercially available kits (Abnova, Jhongli, Taiwan and Abbexa Ltd, Cambridge, United Kingdom, respectively) according to the manufacturers’ instructions.

The avidity of antigen-specific IgGs was evaluated on selected samples with high IgG titers by a modified ELISA^57,58^. Based on the ELISA IgG titers, selected plasma samples were diluted in PBS-1% BSA to obtain an OD of 1 ± 0.5, added (50 μl/well, duplicate wells) to 96-well plates pre-coated with each HPV type-specific VLP, as described above, for 2 h at 37 °C and washed. Negative and positive controls were included in each plate. Plates were then incubated with 50 μl/well of 0.5–5 M GuHCl for each sample (that removes the low avidity antibodies while high avidity antibodies remain bound) for 15 min at room temperature under gentle agitation. Untreated samples and control wells were incubated with PBS alone. After extensive washing, immune complexes were detected as described above. For each sample, the mean OD of duplicates were calculated and percentage of binding was determined as [(treated OD/untreated OD) × 100]. The avidity index (AI) is the extrapolated molar concentration (M) of GuHCl required to reduce the absorbance of the untreated, control well by 50%.

### HPV B-cell Elispot

B-cell Elispot assays were performed using a commercial “Human IgG B-cell Elispot” kit (Mabtech, Nacka Strand, Sweden)^53^. Thawed PBMCs were diluted in RPMI 1640 medium containing 10% FBS (0.5 × 10^6^/ml), seeded in 24-well plates (1 ml/well, duplicate wells), and grown under unstimulated or stimulated conditions with ODN (3 μg/ml), *S. aureus* Cowan I (1:10.000), and Pokeweed (0.1 μg/ml) at 37 °C for 5 days. Ninety-six-well plates (Maipswu, Millipore, Milan, Italy) were pre-washed with 70% ethanol for 2 min and water, and then pre-adsorbed, at 4 °C for 20–24 h, with 100 μl of an anti-human IgG capture antibody (MT91/145; 15 μg/ml) to quantify the total number of B cells secreting IgG, or with 100 μl of each type-specific VLP (12.5 μg/ml) to quantify the number of HPV type-specific B cells. After extensive washing with PBS, 200 μl of unstimulated and stimulated PBMCs (1.25 × 10^6^/ml) were seeded in each HPV type-specific plate (duplicate wells), whereas 200, 100 and 50 μl of unstimulated and stimulated PBMCs (6.25 × 10^4^/ml) were added (duplicate wells) to the total IgG B-cell Elispot wells. In all plates, blank wells containing 200 μl of culture medium alone were included. After incubation at 37 °C for 2 h, plates were washed and processed with an anti-human-IgG-biotin detection antibody provided by the kit. Spots were counted using an Elispot reader (AElvis, Hannover, Germany). The percentage of each HPV type-specific memory B cells was calculated as [antigen-specific B cells (SFC/10^6^ PBMC) / total memory B cells (SFC/10^6^ PBMC)] × 100].

### Flow cytometry

The frequency and phenotype of B and CD4^+^ T lymphocytes were analyzed by flow cytometry. PBMCs were surface stained in the dark for 15 min at room temperature with directly conjugated monoclonal antibodies to identify different B- and T- cell subsets (Supplementary Table 4): eFluor®450-αCD4 and PerCP-Cyanine5.5-αCD45RA (all from eBioscience^TM^-ThermoFisher Scientic); APC-αCD27 and VioBlue®-αCD38 (all from Miltenyi Biotec, Bologna, Italy) and APC/Cyanine7-αCD19 (Biolegend, Koblenz, Germany). Non-viable cells were eliminated from the analysis using LIVE/DEAD Fixable Aqua (Thermo Fisher Scientific). Naïve CD4^+^ T cells were identified as CD27^+^ CD45RA^+^, naïve B cells as CD38^-^ CD27^-^, memory B cells as CD27^+^ CD38^-^ and plasma cells as CD38^high^CD27^+^ (see Supplementary Fig. 4 for representative plots).

To determine glucose uptake, mitochondrial membrane potential, neutral lipid (NL) content or reactive oxygen species (ROS) production, PBMCs were incubated with 50 μM 2′-(N-(7-nitrobenz-2-oxa-1,3-diazol-4-yl)amino)-2-deoxyglucose (2-NBDG) for 20 min at 37 °C, 25 nM tetramethylrhodamine, methyl ester, perchlorate (TMRM) for 30 min at 37 °C, 10 μM 4,4-difluoro-1,3,5,7,8-pentamethyl-4-bora-3a,4a-diaza-*s*-indacene (BODIPY^TM^ 493/503) for 20 min at 37 °C or 5 μM CellROX Green Reagent (all reagents from Thermo Fisher Scientific) for 30 min at 37 °C, respectively^59,60^. Additional stains were used as described above to characterize the metabolic profile of distinct B-cell subsets in each assay. Data were acquired using a BD FACSCanto II flow cytometer (BD Biosciences, Milan, Italy) and analyzed with FlowJo software (BD Biosciences).

### Statistical analysis

Mann–Whitney test was used to compare the difference between two independent groups, while correlations were analyzed by Spearman’s rank test. A simple linear regression was used to estimate the decay (slope) of immune responses (y or Log(y)) as a function of the months post-vaccination (x) for each cohort and age group. The equations of the regression lines have to be intended as y = q + mx or Log(y) = q + mx, with q indicating the intercept and m the slope. Regression lines were compared using ANCOVA. *P*-values less than 0.05 were considered to be statistically significant. Statistical analysis was performed using the softwares R^61^ and Rstudio^62^ with the packages Tidyverse^63^ Scales^64^.

### Reporting summary

Further information on research design is available in the Nature Research Reporting Summary linked to this article.

## Supplementary information

Supplementary Material

REPORTING SUMMARY

## Data Availability

The data that support the findings of this study are available from the corresponding author (A.C.) upon reasonable request.
