# Supplementary material for: Effects of the age of vaccination on the humoral responses to a human papillomavirus vaccine
Source: NPJ Vaccines. 2022 Mar 15;7:37. doi: 10.1038/s41541-022-00458-0 (PMC8924199; doi:10.1038/s41541-022-00458-0)
Supplement: Supplementary file 1 — Supplementary Material [file 41541_2022_458_MOESM1_ESM.pdf]

Supplementary Material

Effects of the age of vaccination on the humoral responses to a human papillomavirus vaccine

Francesco Nicoli[1,2], Barbara Mantelli[2], Eleonora Gallerani[1], Valentina Telatin[2], Laura Squarzon[2], Serena Masiero[2], Riccardo Gavioli[1], Giorgio Palù[2], Luisa Barzon[2], Antonella Caputo[1,2,*]

[1] Department of Chemical, Pharmaceutical and Agricultural Sciences, University of Ferrara, 44121 Ferrara, Italy
[2] Department of Molecular Medicine, University of Padova, 35121 Padova, Italy

[*]Author to whom correspondence should be addressed.

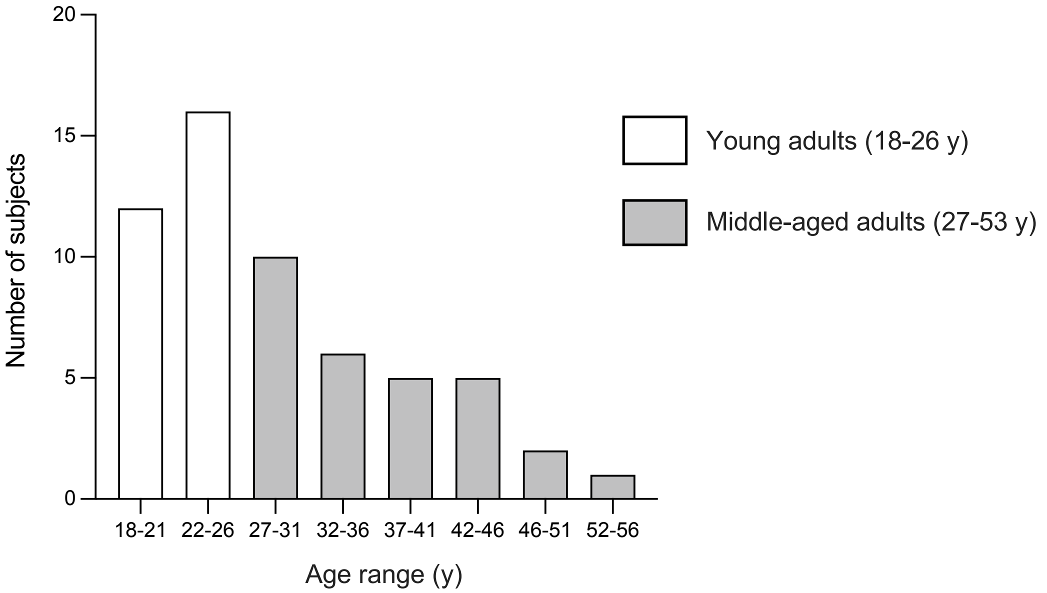

Supplementary Fig. 1. Age stratification of adults of the "Early cohort". Adults of the "Early cohort" were grouped as young adults (18-26 y, n= 28) and middle-aged adults (27-53 y, n=29) and their age-distribution is shown.

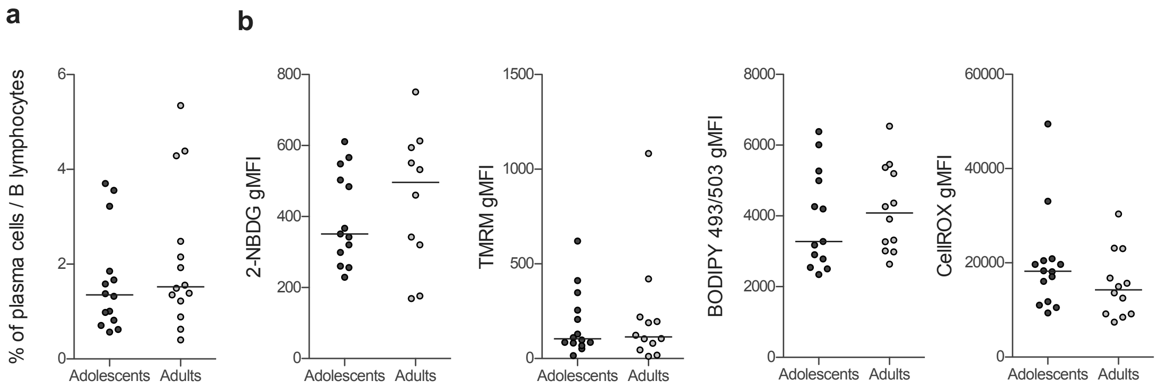

Supplementary Fig. 2. Effect of age on the quantity and quality of plasma cells. (**a**) Percent of plasma cells on total B lymphocytes in adolescents and adults and (**b**) their metabolic properties measured by flow cytometry (glucose uptake with 2-NBDG; mitochondrial membrane potential with TMRM; neutral lipid content with Bodipy 493/503; ROS production with CellROX). Each dot represents a single donor and lines the median; n= 13-14 for adolescents and 10-14 for adults. Samples are from the "Early cohort". Statistical significance was determined by the Mann-Whitney test (all *p*-values were >0.05).

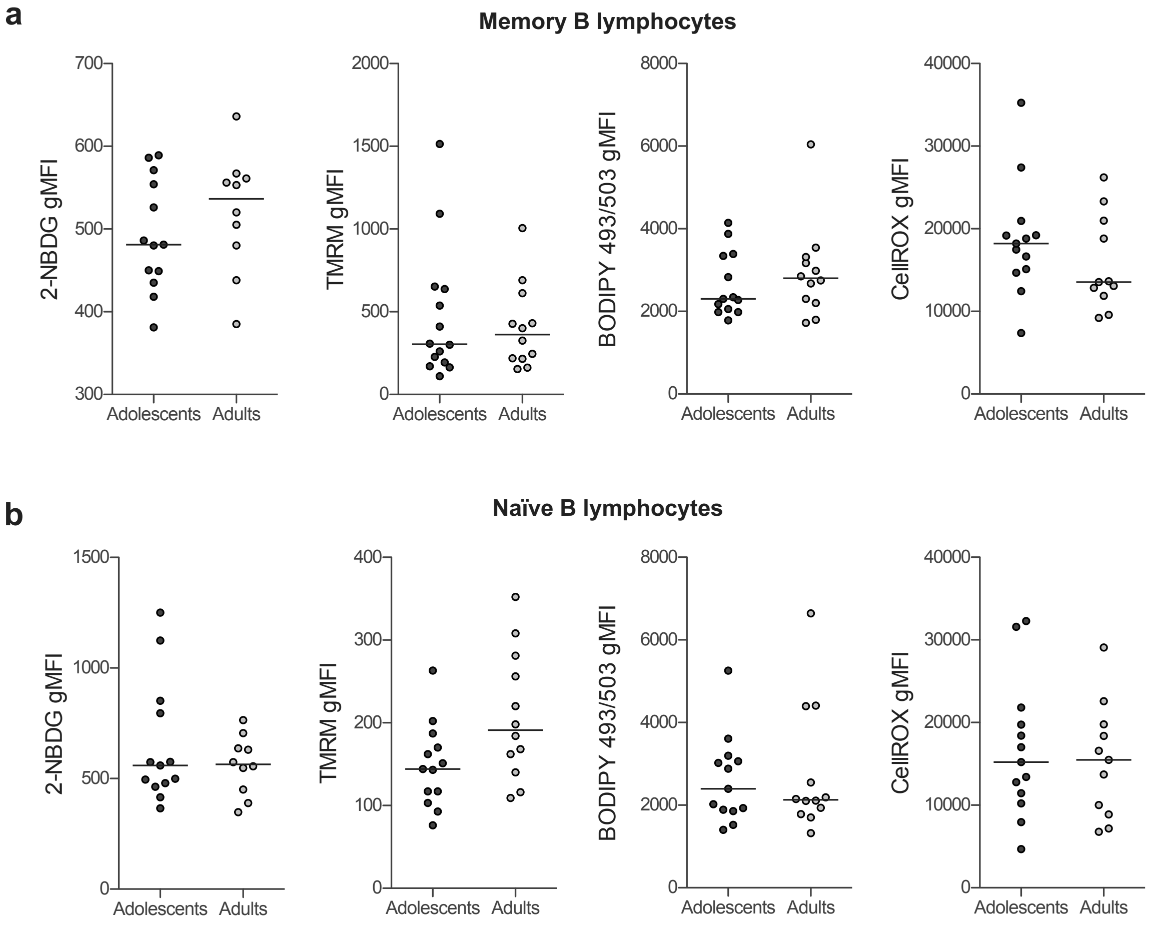

Supplementary Fig. 3. Effect of age on the metabolic properties of memory and naïve B lymphocyte. (**a-b**) Metabolic properties of memory (**a**) and naïve (**b**) B lymphocyte measured by flow cytometry (glucose uptake with 2-NBDG; mitochondrial membrane potential with TMRM; neutral lipid content with Bodipy 493/503; ROS production with CellROX). Each dot represents a single donor and lines the median; n= 13-14 for adolescents and 10-14 for adults. Samples are from the "Early cohort". Statistical significance was determined by the Mann-Whitney test (all *p*-values were >0.05).

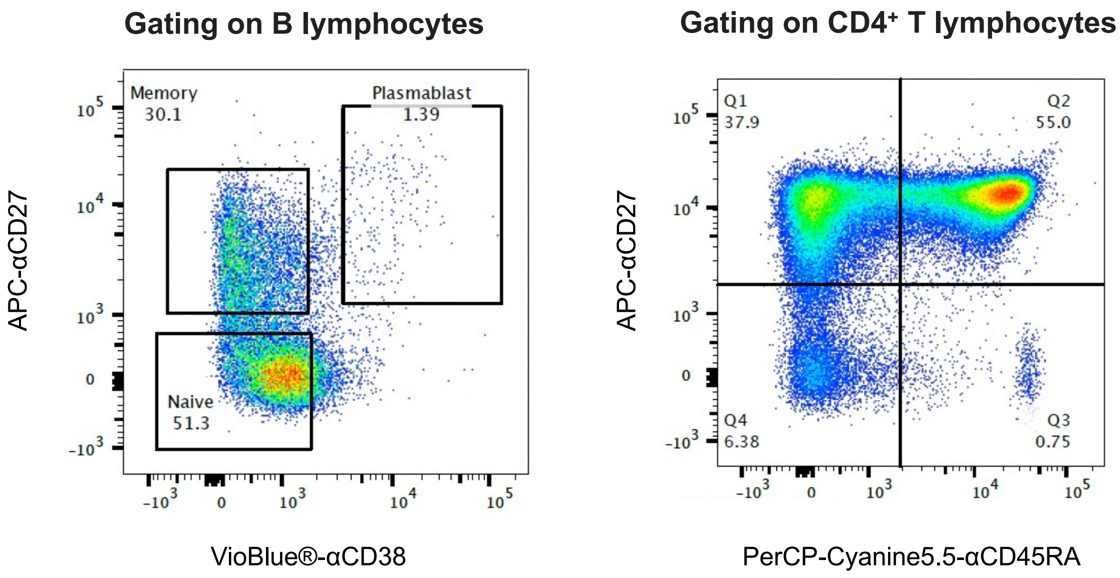

Supplementary Fig. 4. Gating strategy for B- and $CD4^{+}$ T-cell subsets.

Supplementary Table 1. **HPV-specific neutralizing antibodies in adults**

HPV-specific PBNA (ED_{50})	Adults; 18-26 y			Audults, 27-53 y			p value[1]
	Range (min-max)	Median	n	Range (min-max)	Median	n	
HPV-16-specific	160-20480	2560	28	40-20480	2560	29	0.83
HPV-18-specific	1-10240	640	28	1-10240	400	29	0.72

[1]Statistical significance was determined by the Mann-Whitney

Supplementary Table 2. Correlation between HPV-specific and measles/OPV-specific antibody levels

	Correlation with measles-specific antibody index		Correlation with the optical density of OPV-specific antibodies	
	Spearman's r	Spearman's p value	Spearman's r	Spearman's p value
HPV-6-specific IgG titers	0.23	0.20	0.19	0.27
HPV-11-specific IgG titers	-0.01	0.97	0.27	0.11
HPV-16-specific IgG titers	0.09	0.64	0.25	0.13
HPV-18-specific IgG titers	0.43	0.05	0.26	0.13

Supplementary Table 3. Correlation between HPV-specific antibody levels and the PC or memory B lymphocyte frequencies

HPV-specific early responses	Correlation with the % of PC over total B lymphocytes		Correlation with the % of memory over total B lymphocytes	
	Spearman's r	Spearman's p value	Spearman's r	Spearman's p value
HPV-6-specific IgG titers	-0.13	0.56	-0.11	0.62
HPV-11-specific IgG titers	-0.16	0.46	-0.28	0.20
HPV-16-specific IgG titers	-0.15	0.51	-0.24	0.28
HPV-18-specific IgG titers	-0.20	0.36	-0.23	0.30

Supplementary Table 4. Directly conjugated antibodies used for flow cytometry staining

Antibody	Fluorochrome	Supplier and reference	Amount used (µl)/tube
CD27	APC	MILTENYI BIOTEC 130-113-626	1.5
CD38	VioBlue®	MILTENYI BIOTEC 130-110-445	3
CD4	eFluor®450	EBIOSCIENCES 48-0042-82	2
CD45RA	PerCP-Cy5.5	EBIOSCIENCES 45-0458-42	3
CD19	APC-Cy7	BIOLEGEND 302218	1
