# Supplementary material for: Effects of the age of vaccination on the humoral responses to a human papillomavirus vaccine
Source: NPJ Vaccines. 2022 Mar 15;7:37. doi: 10.1038/s41541-022-00458-0 (PMC8924199; doi:10.1038/s41541-022-00458-0)
Supplement: Supplementary file 2 — REPORTING SUMMARY [file 41541_2022_458_MOESM2_ESM.pdf]

nature portfolio

Corresponding author(s): Antonella Caputo

Last updated by author(s): Sep 29, 2021

Reporting Summary

Nature Portfolio wishes to improve the reproducibility of the work that we publish. This form provides structure for consistency and transparency in reporting. For further information on Nature Portfolio policies, see our Editorial Policies and the Editorial Policy Checklist.

Statistics

For all statistical analyses, confirm that the following items are present in the figure legend, table legend, main text, or Methods section.

n/a	Confirmed	
☐	☒	The exact sample size (*n*) for each experimental group/condition, given as a discrete number and unit of measurement
☐	☒	A statement on whether measurements were taken from distinct samples or whether the same sample was measured repeatedly
☐	☒	The statistical test(s) used AND whether they are one- or two-sided<br>*Only common tests should be described solely by name; describe more complex techniques in the Methods section.*
☒	☐	A description of all covariates tested
☒	☐	A description of any assumptions or corrections, such as tests of normality and adjustment for multiple comparisons
☐	☒	A full description of the statistical parameters including central tendency (e.g. means) or other basic estimates (e.g. regression coefficient) AND variation (e.g. standard deviation) or associated estimates of uncertainty (e.g. confidence intervals)
☐	☒	For null hypothesis testing, the test statistic (e.g. *F*, *t*, *r*) with confidence intervals, effect sizes, degrees of freedom and *P* value noted<br>*Give P values as exact values whenever suitable.*
☒	☐	For Bayesian analysis, information on the choice of priors and Markov chain Monte Carlo settings
☒	☐	For hierarchical and complex designs, identification of the appropriate level for tests and full reporting of outcomes
☐	☒	Estimates of effect sizes (e.g. Cohen's *d*, Pearson's *r*), indicating how they were calculated

Our web collection on statistics for biologists contains articles on many of the points above.

Software and code

Policy information about availability of computer code

Data collection	Magellan was used to collect ELISA data, FACS DIVA was used to collect Flow cytometry data, Eli.Analyse was used to collect Elispot data.
Data analysis	Statistical analysis was performed using the softwares R and Rstudio with the packages Tidyverse Scales. FlowJo was used to analyse flow cytometry data.

For manuscripts utilizing custom algorithms or software that are central to the research but not yet described in published literature, software must be made available to editors and reviewers. We strongly encourage code deposition in a community repository (e.g. GitHub). See the Nature Portfolio guidelines for submitting code & software for further information.

Data

Policy information about availability of data

All manuscripts must include a data availability statement. This statement should provide the following information, where applicable:

- Accession codes, unique identifiers, or web links for publicly available datasets
- A description of any restrictions on data availability
- For clinical datasets or third party data, please ensure that the statement adheres to our policy

The data that support the findings of this study are available from the corresponding author (A.C.) upon reasonable request.

Field-specific reporting

Please select the one below that is the best fit for your research. If you are not sure, read the appropriate sections before making your selection.

☒ Life sciences ☐ Behavioural & social sciences ☐ Ecological, evolutionary & environmental sciences

For a reference copy of the document with all sections, see nature.com/documents/nr-reporting-summary-flat.pdf

Life sciences study design

All studies must disclose on these points even when the disclosure is negative.

Sample size	Power calculations depend on the assay. The total sample size (recruited donors) largely overcomes the minimum number of required donors per group and was based on feasibility factors. The same applies for each type of assay. We used statistics appropriate to the data being presented.
Data exclusions	No data were excluded, to take into account the variability between individuals.
Replication	We have internal replicates where appropriate (ELISA, ELIspot, Avidity and PBNA). All the data presented are the independent biological replicates.
Randomization	N/A
Blinding	N/A

Reporting for specific materials, systems and methods

We require information from authors about some types of materials, experimental systems and methods used in many studies. Here, indicate whether each material, system or method listed is relevant to your study. If you are not sure if a list item applies to your research, read the appropriate section before selecting a response.

Materials & experimental systems

n/a	Involved in the study	
☐	☒	Antibodies
☒	☐	Eukaryotic cell lines
☒	☐	Palaeontology and archaeology
☒	☐	Animals and other organisms
☐	☒	Human research participants
☒	☐	Clinical data
☒	☐	Dual use research of concern

Methods

n/a	Involved in the study	
☒	☐	ChIP-seq
☐	☒	Flow cytometry
☒	☐	MRI-based neuroimaging

Antibodies

Antibodies used	eFluor®450-αCD4 and PerCP-Cyanine5.5-αCD45RA (all from eBioscienceTM-ThermoFisher Scientic); APC-αCD27 and VioBlue®-αCD38 (all from Miltenyi Biotec, Bologna, Italy) and APC/Cyanine7-αCD19 (Biolegend, Koblenz, Germany).
Validation	All antibodies used are validated by the manufacturer for use on human cells and internally for use on human PBMCs. All antibodies and widely used in literature.

Human research participants

Policy information about studies involving human research participants

Population characteristics	The population was composed of 315 female subjects. One subgroup (n=181) was tested at 1 to 8 months (median of 2 months) after the third vaccine dose and is defined as "Early cohort", while a second subgroup (n=153) was tested at 1 to 5 years (median of 4 years) after the third vaccine dose and is defined as "Late cohort". In the "Early cohort", 117 were adolescents at the time of vaccination (age range: 10-14-year-old, median age: 11y) and 57 were adults (age range: 18-53-year-old, median age: 26y). In the "Late cohort", 70 were adolescents at the time of vaccination (age range: 11-14-year-old, median age: 12y) and 71 were adults (age range: 18-26-year-old, median age: 21y).
Recruitment	Enrolment was carried out at the public health district of Padova (Veneto region, Italy) where HPV vaccine was offered by organized vaccination (10-14 years old) and implementation (older ages) programs. Girls and women who had previously completed the vaccination schedule were invited by letter to participate in the study.

Ethics oversight | Ethics Committee of Padova University-Hospital (Protocol no 2413P)

Note that full information on the approval of the study protocol must also be provided in the manuscript.

Flow Cytometry

Plots

Confirm that:

☒ The axis labels state the marker and fluorochrome used (e.g. CD4-FITC).

☒ The axis scales are clearly visible. Include numbers along axes only for bottom left plot of group (a 'group' is an analysis of identical markers).

☒ All plots are contour plots with outliers or pseudocolor plots.

☒ A numerical value for number of cells or percentage (with statistics) is provided.

Methodology

Sample preparation	PBMCs were surface stained in the dark for 15 min at room temperature with directly conjugated monoclonal antibodies to identify different B and T cell subsets.
Instrument	BD FACSCanto II flow cytometer
Software	FACS Diva and FlowJo
Cell population abundance	For flow cytometry analysis a minimum of 10,000 B cells cells were recorded.
Gating strategy	Singlets are gated on FSC-A/FSC-H. Global cell population is gated on FSC-A/SSC-A. Live cells - using Aqua LIVE/DEAD are gated. Naïve CD4+ T cells were identified as CD27+ CD45RA+, naïve B cells as CD38- CD27-, memory B cells as CD27+ CD38- and plasma cells as CD38highCD27+.

☒ Tick this box to confirm that a figure exemplifying the gating strategy is provided in the Supplementary Information.
